# A benchmark study of convolutional neural networks in fully automatic segmentation of aortic root

**DOI:** 10.3389/fbioe.2023.1171868

**Published:** 2023-06-15

**Authors:** Tingting Yang, Guangyu Zhu, Li Cai, Joon Hock Yeo, Yu Mao, Jian Yang

**Affiliations:** ^1^ School of Energy and Power Engineering, Xi’an Jiaotong University, Xi’an, China; ^2^ School of Mathematics and Statistics, Northwestern Polytechnical University, Xi’an, China; ^3^ School of Mechanical and Aerospace Engineering, Nanyang Technological University, Singapore, Singapore; ^4^ Department of Cardiac Surgery, Xijing Hospital, The Fourth Military Medical University, Xi’an, China

**Keywords:** TAVR, aortic root, automatic segmentation, 3D CNN, deep learning

## Abstract

Recent clinical studies have suggested that introducing 3D patient-specific aortic root models into the pre-operative assessment procedure of transcatheter aortic valve replacement (TAVR) would reduce the incident rate of peri-operative complications. Tradition manual segmentation is labor-intensive and low-efficient, which cannot meet the clinical demands of processing large data volumes. Recent developments in machine learning provided a viable way for accurate and efficient medical image segmentation for 3D patient-specific models automatically. This study quantitively evaluated the auto segmentation quality and efficiency of the four popular segmentation-dedicated three-dimensional (3D) convolutional neural network (CNN) architectures, including 3D UNet, VNet, 3D Res-UNet and SegResNet. All the CNNs were implemented in PyTorch platform, and low-dose CTA image sets of 98 anonymized patients were retrospectively selected from the database for training and testing of the CNNs. The results showed that despite all four 3D CNNs having similar recall, Dice similarity coefficient (DSC), and Jaccard index on the segmentation of the aortic root, the Hausdorff distance (HD) of the segmentation results from 3D Res-UNet is 8.56 ± 2.28, which is only 9.8% higher than that of VNet, but 25.5% and 86.4% lower than that of 3D UNet and SegResNet, respectively. In addition, 3D Res-UNet and VNet also performed better in the 3D deviation location of interest analysis focusing on the aortic valve and the bottom of the aortic root. Although 3D Res-UNet and VNet are evenly matched in the aspect of classical segmentation quality evaluation metrics and 3D deviation location of interest analysis, 3D Res-UNet is the most efficient CNN architecture with an average segmentation time of 0.10 ± 0.04 s, which is 91.2%, 95.3% and 64.3% faster than 3D UNet, VNet and SegResNet, respectively. The results from this study suggested that 3D Res-UNet is a suitable candidate for accurate and fast automatic aortic root segmentation for pre-operative assessment of TAVR.

## 1 Introduction

Transcatheter aortic valve replacement (TAVR) is a revolutionary technology for treating aortic stenosis (AS), with the advantages of no thoracotomy, less trauma, and faster recovery than surgical valve replacement (Elattar et al., 2014; Eleid and Holmes, 2017). With the emerging trend of expanding TAVR indications to younger and low-surgical-risk AS patients, the safety of TAVR has become a universal concern which requires careful patient-specific pre-operative planning and assessment (Walther et al., 2015; Leon et al., 2016; Puri et al., 2017; Youssefi et al., 2017; Mack et al., 2019). In an attempt to gap the limitation of the medical imaging assessment, recent developments provide powerful tools to support clinicians for precise patient-specific pre-operative morphological and functional assessment, such as image-based patient-specific 3D reconstruction, 3D printing, virtual reality (VR), augmented reality (AR) and numerical simulation techniques represented by finite element analysis (FEA) and computational fluid dynamics (CFD) (Levin et al., 2020; Jian et al., 2021; Otto et al., 2021; Wang et al., 2021; Chessa et al., 2022; Li et al., 2022).

As the basis of these applications, the rapid and accurate segmentation and 3D reconstruction of medical images have gradually become the key issues for personalized medicine. Traditional segmentation procedures mainly rely on manual operations requiring specialist knowledge, which are still time-consuming and labor-intensive (Kasel et al., 2013; Maragiannis et al., 2014; De Jaegere et al., 2016; Qian et al., 2017; Haghiashtiani et al., 2020). Especially when it comes to pre-operative TAVR assessment, manual segmentation generally cost tens of minutes due to the complex anatomy of the aortic root, including the ascending aorta, aortic valve, and coronary arteries. Thus, manual segmentation cannot provide a time-sensitive clinical recommendation with the increasingly large scale of patients receiving TAVR and the urgency of TAVR when patients with severe AS occasionally present with an acute decompensated state (Elbadawi et al., 2020). Moreover, the quality and reproducibility of manual segmentation are difficult to guarantee, and human errors induced in the manual segmentation stage could result in inaccurate or even wrong analysis results (Bertolini et al., 2022). These drawbacks prohibit its daily application in clinical centers.

To address the difficulties mentioned above, studies on the automatic segmentation of medical images started in the middle of the 1990s. The early stage of automatic image segmentation is featured by the methods based on supervised techniques such as active shaped models, which still require human intervention in extracting discriminant features from the images (Cootes et al., 1995; Cribier, 2002; Bengio et al., 2013; Kenny and Monaghan, 2015). With the growing clinical demand and the development of artificial intelligence techniques, fully automatic medical image segmentation methods based on deep learning (DL) overcame the drawbacks mentioned above and have become the technique of choice over the past decade (Smith et al., 2011; Litjens et al., 2017; Shen et al., 2017; Minaee et al., 2020). Among the segmentation-dedicated DL methods, 3D CNNs based architectures that were capable of analyzing volumetric medical image data provided powerful tools for the 3D reconstruction of lesions and organs (Thalji et al., 2014; Ranschaert et al., 2019; Panayides et al., 2020; Tsakanikas et al., 2020; Banerjee et al., 2021; Romaszko et al., 2021; Harrison et al., 2022). Among these, 3D UNet was the first medical image segmentation-dedicated 3D CNN, which was proposed in 2016 (Çiçek et al., 2016). It was first used to segment kidneys and then quickly extended to other tissues or organs, such as cardiac, brain and lung (Lundervold and Lundervold, 2019; Chen et al., 2020; Liu et al., 2021a). Since then, several novel 3D CNNs have been proposed for medical image segmentation, including VNet, variants of 3D UNet (3D Res-UNet, 3D Dense-UNet, 3D Attention-UNet, etc.), SegResNet, etc (Milletari et al., 2016; Fang et al., 2019; Myronenko, 2019; Liu et al., 2021b).

In recent years, these 3D CNNs have been gradually used to segment the aortic root automatically (Fassa et al., 2013; Fan et al., 2019; Ravichandran et al., 2019; Macruz et al., 2022; Sieren et al., 2022). Sieren et al. compared the performance between manual and 3D UNet segmentation based on CTA exams of the aorta of 191 patients and demonstrated that automated aorta segmentation by using 3D UNet is feasible (Sieren et al., 2022). Macruz et al. presented a 3D UNet-based framework for automated segmentation of the thoracic aorta in thoracic CT studies, which provides the basis for determining aortic diameter measurements and accurately predicting thoracic aortic aneurysms (Macruz et al., 2022). Ravichandran et al. evaluated the performance of 3D UNet and its variants in 3D segmentation of the aortic root under small samples by using a single evaluation metric Dice similarity coefficient (DSC), which indicated that the segmentation quality of the variants of 3D UNet is better than that of 3D UNet (Ravichandran et al., 2019). Although the current state-of-the-art literature focusing on CNN-based segmentation of aortic root is promising, there is a lack of a comprehensive evaluation of both segmentation quality and efficiency of the current popular 3D CNN architectures, especially for TAVR procedure which requires rapid and accurate pre-operative assessment.

Therefore, in this study, we compared the comprehensive segmentation performance of the four popular segmentation-dedicated 3D CNNs (3D UNet, VNet, 3D Res-UNet and SegResNet) under small sample low-dose CT datasets and realized a fully automated, accurate segmentation and reconstruction framework, which provides a reliable guarantee for pre-operative morphological and functional assessment of TAVR.

## 2 Materials and methods

### 2.1 Data preparation

#### 2.1.1 Imaging data

In this study, 98 sets of anonymized chest CTA images acquired from low-dose multidetector 128-slice CT scanners (uCT 760, United Imaging Healthcare, Shanghai, China) were retrospectively collected from the pre-operative TAVR examination database of patients without calcification. All the scans included the aorta with 192–339 slices in the *Z*-axis. The slice size, thickness, and tube voltage are 512 × 512 pixels, 0.75 mm, and 120 kV, respectively. The pixel spacing of the scans varied between 0.25 and 0.60 mm. The axial spacing between slices is 0.5 mm. The image sets in Digital Imaging and Communications in Medicine (DICOM) format were manually inspected, and three image sets were excluded due to low signal-noise ratio or severe artifacts.

#### 2.1.2 Label annotation

**FIGURE 1:**
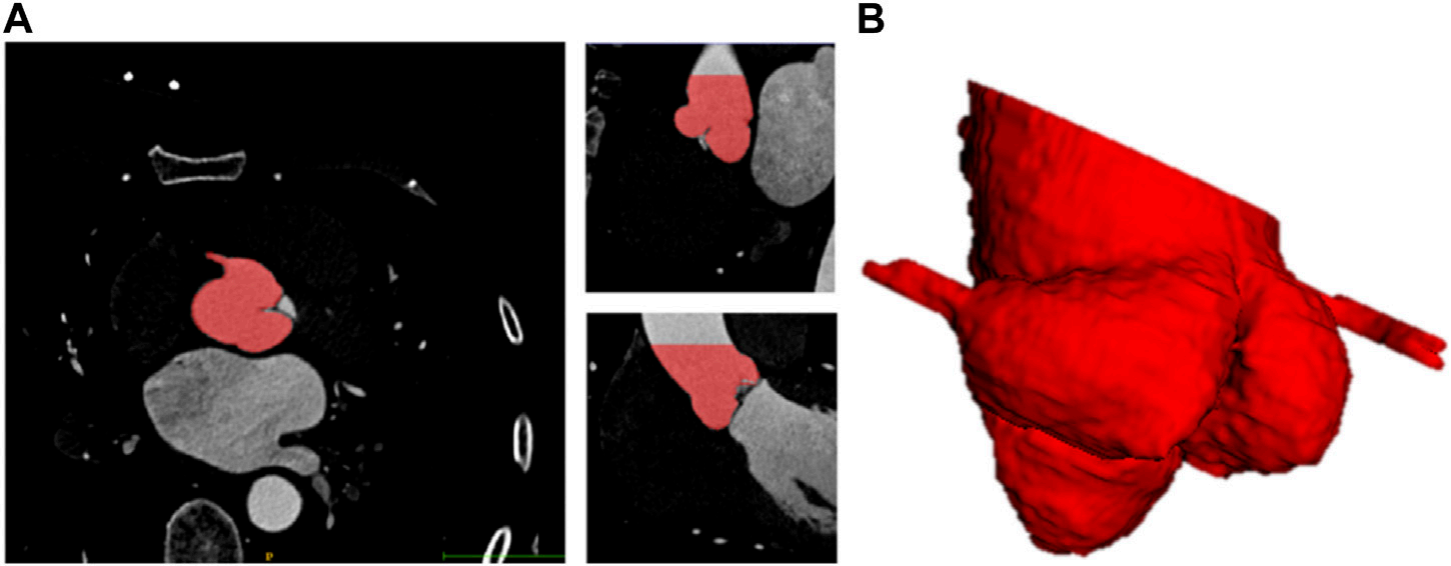
Schematic diagram of 3D label of aortic root. **(A)** Sagittal, coronal, horizontal view of label. **(B)** 3D visualization of label of aortic root.

To reduce human error, two clinical engineers experienced in image segmentation and a senior radiologist participated in the labeling process. Firstly, the patient CT data in DICOM format were imported into Materialise Mimics version 21.0 software (Materialise, Leuven, Belgium) and three orthogonal sections were manually established based on the aortic annulus plane using the multiplane reconstruction function of the software (Ya’qoub et al., 2022). After that, the engineers individually labeled the mask of the aortic root with the help of the threshold segmentation algorithm (Underwood et al., 2000) (Figure 1). The mask covered the entire aortic root, an approximately 40 mm long segment starting from the aortic annulus plane. What’s more, the position of the coronary ostium is crucial for pre-operative assessment of TAVR to avoid coronary obstruction. Therefore, the coronary arteries about 10 mm distal from the coronary ostium were included in the mask as well. The average manual mask time for each patient is about 26 min. After the label annotation, the senior radiologist decides the label to be used in the experiments and the ground truth based on the quality assessment.

#### 2.1.3 Image pre-processing

Due to the original CTA images being too large and noisy, a series of data pre-processing is conducted before training and validation. Firstly, noise reduction and normalization of the images were performed to enhance the signal-to-noise ratio of the images. Next, the pixel and axial spacing were adjusted to 1 and 0.75 using bilinear interpolation to compress the data. In addition, to increase data diversity and improve training robustness, data augmentation was conducted on the pre-processed images following the methods described by Chlap et al. (2021) and He et al. (2011). All the volume data in the training set were augmented by randomly cropping into four sub-volumes, with the center being a foreground or background voxel based on the Positive-to-Negative Ratio of 1:1. Especially the size of the cropped sub-volumes used in this study was selected by the pre-tests which compared the segmentation performance of the three different sub-volume sizes (64 × 64 × 64 pixels, 96 × 96 × 96 pixels, 128 × 128 × 128 pixels) on the validation set. After the augmentation, the original training set size was multiplied four times.

### 2.2 CNN architectures for evaluation

Four 3D CNN architectures were evaluated in this study, including 3D UNet, 3D Res-UNet, VNet, and SegResNet. The specific differences between the evaluated 3D CNN architectures are listed in Table 1.

**TABLE 1:** Model architecture comparison of different 3D CNNs.

Networks	Params/M	Layers	Kernel size	Residual block	Up/down sampling	Skip connection
3D UNet	5.70	5	3 × 3 × 3		Max pooling	concat
VNet	45.60	5	5 × 5 × 5	√	convolution	add
3D Res-UNet	4.81	5	3 × 3 × 3	√	convolution	concat
SegResNet	1.17	4	3 × 3 × 3	√	convolution	add

#### 2.2.1 3D UNet model

The 3D UNet model is a popular architecture based on 3D CNN, which consists of an encoder path and a decoder path (Çiçek et al., 2016). The 3D UNet used in this study has five layers with 16, 32, 64, 128 and 256 feature channels, respectively. In the encoder path, each layer contains two 3 × 3 × 3 convolutions, and then a 2 × 2 × 2 max pooling with strides of two in each dimension. In the decoder path, each layer consists of an up-convolution of 2 × 2 × 2 by strides of two in each dimension, followed by two 3 × 3 × 3 convolutions. The shortcut connections of equal-resolution layers in the encoder path provide high-resolution features for the decoder path. Instance normalization is used to prevent contrast shifting, ensuring input image contrast is not skewed by being batched with images with significantly different contrast ranges.

#### 2.2.2 VNet model

The VNet model is another widely used architecture for 3D medical image segmentation (Milletari et al., 2016). In this architecture, a residual block was added to each layer, and the pooling operations in 3D UNet were replaced by 2 × 2 × 2 strided convolutions. In addition, the channel concatenation in the skip connections of 3D UNet was replaced with the element-wise summation. Benefiting from replacing pooling with convolution, VNet not only reduces memory demand during training but also makes the training process better understood and analyzed. In addition, the use of residual block alleviates the gradient vanishing problem in the deep networks. In this study, the VNet has 5 layers. The residual block in the first, second, and rest layers contain one, two, and three 5 × 5 × 5 convolutions, respectively.

#### 2.2.3 3D Res-UNet

The 3D Res-UNet model is one of the most famous derivations of the 3D UNet model. Compared with 3D UNet, 3D Res-UNet improved the accuracy and efficiency in image segmentation by introducing residual block into the down-sampling path and up-sample unit in the up-sampling path (Yang et al., 2019). It also applied the 2 × 2 × 2 convolutions to replace the pooling operation. Furthermore, parametric rectifying linear units allow the network to learn a better activation, improving segmentation performance.

#### 2.2.4 SegResNet

The SegResNet model was recently proposed and performed well in 3D MRI brain tumor segmentation as an encoder-decoder-based asymmetrical 3D CNN architecture with a larger encoder to extract image features and a smaller decoder to segment the image (Myronenko, 2019). Each encoder layer has a different number of ResNet-like blocks. Different from other networks, each decoder layer first begins with upsizing to reduce the number of features by using 1 × 1 × 1 convolution and double the spatial dimension by using 3D bilinear up-sampling, followed by the addition encoder output of the equivalent spatial level.

### 2.3 Training and testing

#### 2.3.1 Implementation details

All the 3D CNNs were trained for 500 epochs using the Adam optimizer with the following parameters: learning rate = 0.0001, *β*_1_ = 0.9, *β*_2_ = 0.999, *ε* = 1e-8, batch size = 1. The Dice coefficient loss function (Diceloss) is used as loss function to evaluate the convergency of the training in all experiments. The definition of Diceloss is given in Eq. 1, where *y*_*gt*_ and *y*_*pred*_ are the ground truth and binary predictions from CNNs, ε was a constant set to 1e-5.

$$Diceloss=1-\frac{2\left|y_{gt}\cap y_{pred}\right|+\varepsilon }{\left|y_{gt}\right|+\left|y_{pred}\right|+\varepsilon }$$ (1)

The entire dataset was randomly divided into a training set (76 images) and a validation set (19 images). To explore the impact of the size of the training set on the segmentation performance, a sensitivity analysis was first conducted by setting up the three different sizes of the training set, 25%, 50% and 100% of the entire training set, respectively. After that, a five-fold cross-validation scheme was adopted for each 3D CNN under the entire training set. Due to the large original image, a 160 × 160 × 160 pixels sliding window is used to divide the validation set images to improve computing efficiency.

**FIGURE 2:**
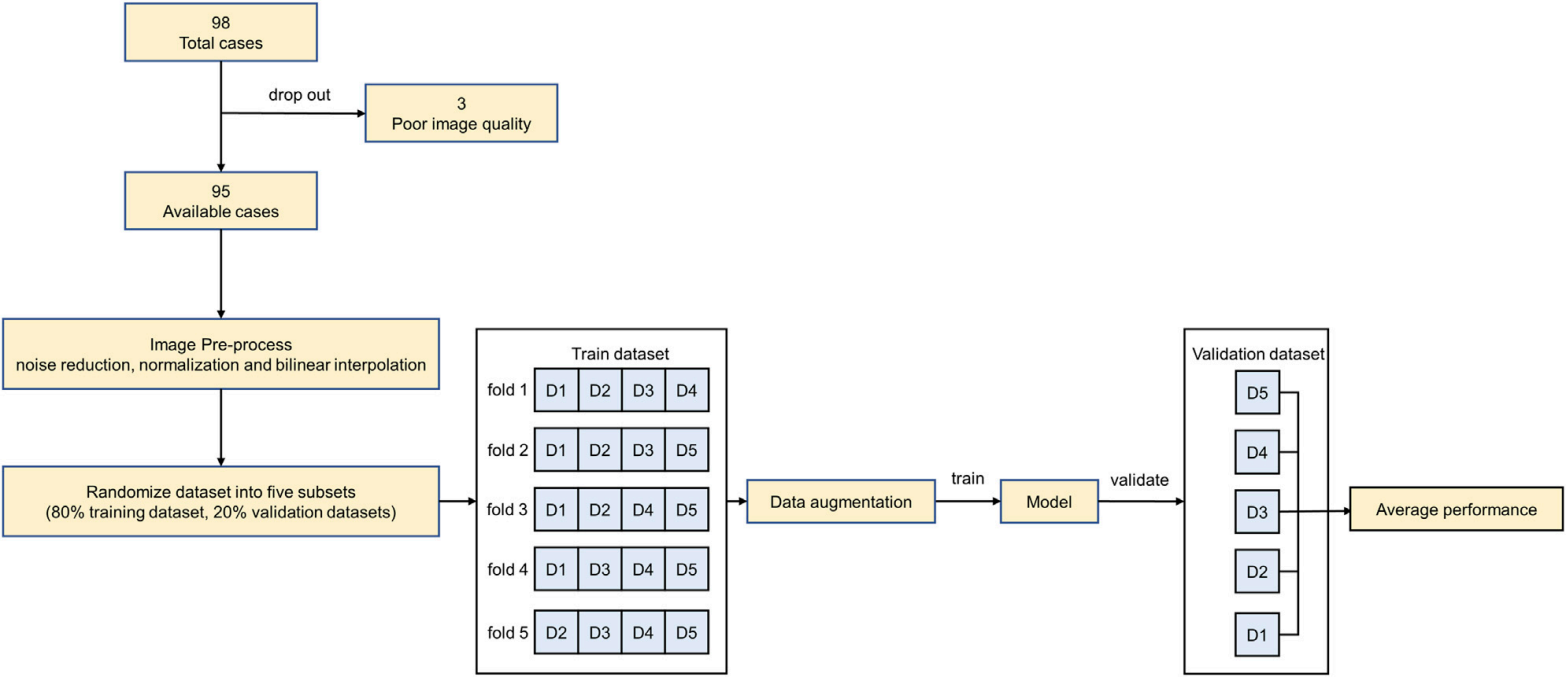
The overall experimental design of the workflow diagram.

All experiments were implemented on a workstation powered by an NVIDIA GeForce RTX 3090 GPU with 24 GB of RAM. The code was implemented with PyTorch 1.9.1 in Windows 10. The overall experimental design of the workflow diagram is shown in Figure 2.

#### 2.3.2 Evaluation metrics

To quantitively compare the segmentation performances of the 3D CNNs, five metrics widely used in segmentation quality assessment were evaluated in this study, including recall, Dice similarity coefficient (DSC), Jaccard index, Hausdorff distance (HD) and 3D deviation. The specific definitions of the metrics are listed below.

Recall, also called Sensitivity or True Positive Rate (TPR), measures the portion of positive voxels in the ground truth identified as positive by the predictions of CNNs (Eq. 2).

$$Recall=\frac{y_{gt}\cap y_{pred}}{y_{gt}}$$ (2)

DSC is the pair-wise overlap ratio between the predictions and the ground truth, ranging from 0 to 1 (Eq. 3).

$$DSC=\frac{2\left|y_{gt}\cap y_{pred}\right|}{\left|y_{gt}\right|+\left|y_{pred}\right|}$$ (3)

Jaccard index also reflects the overlap ratio between the intersection and union of predictions and the ground truth with the range between 0 and 1 (Eq. 4).

$$Jaccard=\frac{\left|y_{gt}\cap y_{pred}\right|}{\left|y_{gt}\cup y_{pred}\right|}$$ (4)

HD measures the distance between *y*_*gt*_ and *y*_*pred*_ by indicating the greatest distances from a point in *y*_*pred*_ to the closest point in *y*_*gt*_ (Eq. 5).

$$HD=\max\left\{d_{y_{gt}y_{pred}},d_{y_{pred}y_{gt}}\right\}=\max\left\{\max_{x\in y_{gt}}\min_{y\in y_{pred}}d\left(x,y\right),\max_{y\in y_{pred}}\min_{x\in y_{gt}}d\left(x,y\right)\right\}$$ (5)

The CNN-based segmentation results and ground truth were 3D reconstructed using the Python platform and VTK library. The 3D deviation metrics between CNN-based segmentation and ground truth were analyzed using Geomagic software (Geomagic Inc., Research Triangle Park, NC). In addition, the segmentation efficiency of the 3D CNNs was compared by using the average segmentation time spent on the validation set.

## 3 Results

### 3.1 Impacts of sub-volume size on model performance

**FIGURE 3:**
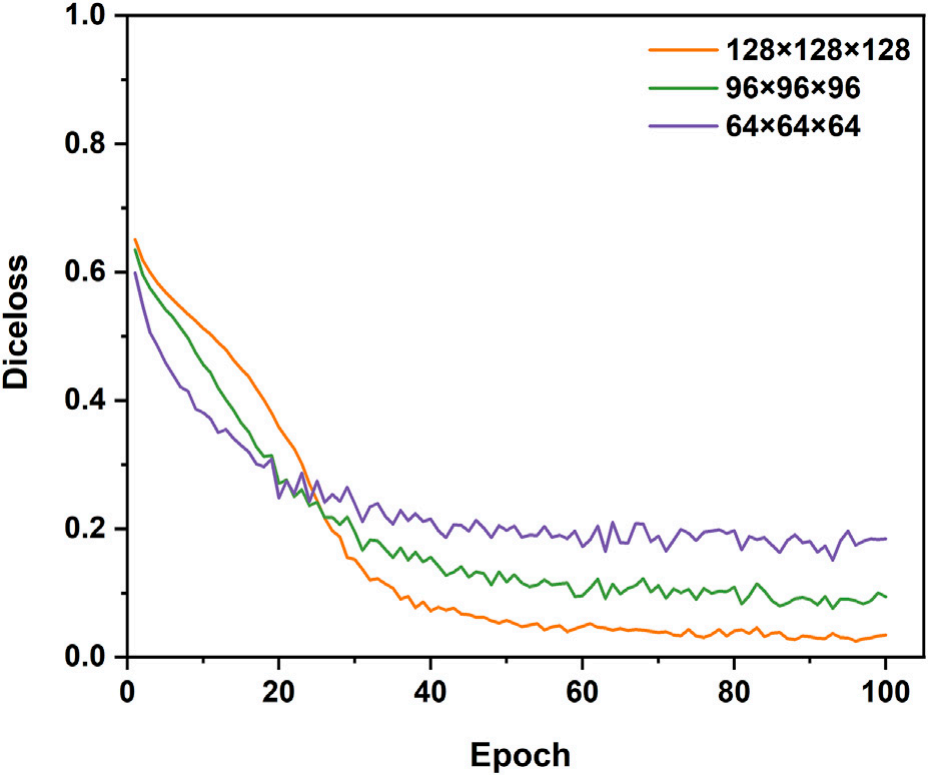
The influence of different sizes of sub-volumes on the convergence of the loss function during training.

**FIGURE 4:**
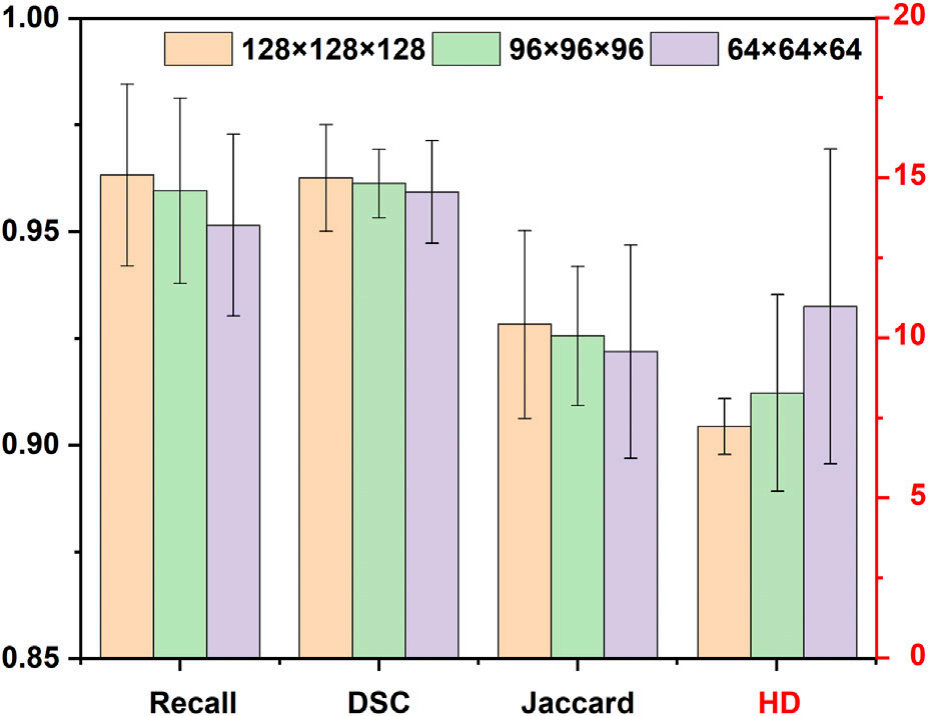
The influence of different sizes of sub-volumes on the segmentation performance of validation set samples.

To explore the suitable sub-volume size of the training set for our study, the pre-tests of the impacts of sub-volume size on 3D CNNs performance were conducted. The three different sub-volume sizes (64 × 64 × 64 pixels, 96 × 96 × 96 pixels, 128 × 128 × 128 pixels) of the training set were selected to train and validate 3D Res-UNet for just one fold under the entire training set. Subject to hardware limitation, the sub-volume size of 128 × 128 × 128 pixels was the largest size implemented to train 3D Res-UNet in this study. The results showed that for these three sub-volume sizes, the convergence rate was negatively related to the sub-volume size, while segmentation quality performance is positively related to that (Figure 3) (Figure 4). This might indicate that the larger sub-volume size would produce better segmentation quality. In the subsequent studies, 304 (76 × 4) augmented 128 × 128 × 128 pixels sub-volumes were employed to train different 3D CNNs.

### 3.2 Impacts of training dataset sizes on model performance

#### 3.2.1 Training performance

**FIGURE 5:**
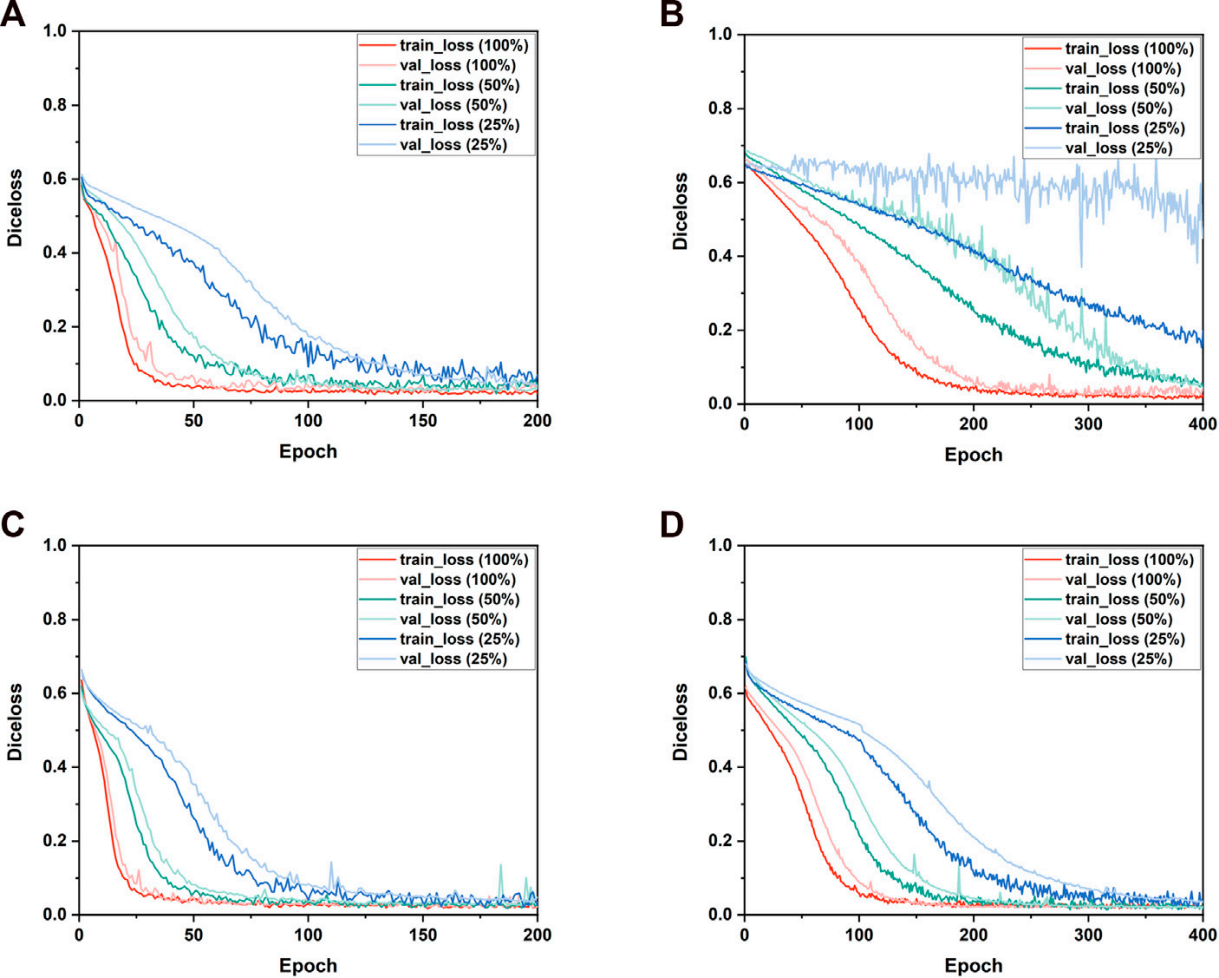
The performance of the training and validation process. The changes of the Diceloss of **(A)** 3D UNet, **(B)** VNet, **(C)** 3D Res-UNet and **(D)** SegResNet during training and validation process under different training set sizes.

Figure 5 shows the Diceloss profiles of training and validation under different sizes of the training set. It demonstrated that the convergence rate of all the 3D CNNs was positively related to the size of the training set. When trained by 25% of the entire training set, only VNet performed a notable overfitting phenomenon which might be caused by its higher model complexity and the limited dataset. When trained by 100% of the entire training set, the training set error and the validation set error of 3D CNNs had the same downward trend, indicating that all 3D CNNs found a good fit between under-fitting and over-fitting in the training process. Therefore, the subsequent five-fold validation was conducted to compare the segmentation performance of the four 3D CNNs using the entire training set and the validation set (Section 3.3). In addition, compared with VNet and SegResNet, 3D UNet and 3D Res-UNet performed a faster convergence rate by reaching a training Diceloss of nearly 0.15 in less than 50 epochs with 100% of the entire training set.

#### 3.2.2 Segmentation performance

**FIGURE 6:**
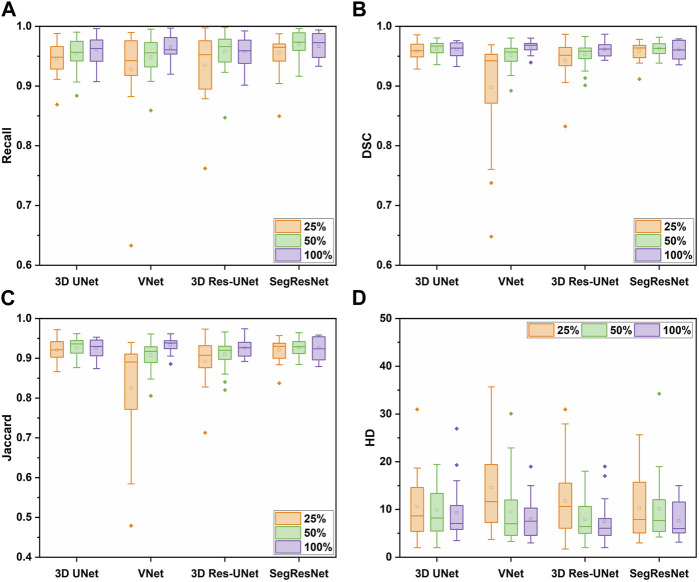
The segmentation performance of the four 3D CNNs trained by different size of the training set, including **(A)** Recall, **(B)** DSC, **(C)** Jaccard, and **(D)** HD metrics.

Figure 6 shows the segmentation performance of the 3D CNNs under the different sizes of the training sets. The segmentation performance of all the 3D CNNs was affected by the size of the training dataset. Among these 3D CNNs, the segmentation quality of VNet is more sensitive to the size of the training set. Table 2 showed that the evaluation metrics notably deteriorated with the decrease of training set size in VNet, while the performance deterioration in other CNNs is mild.

**TABLE 2:** The segmentation quality of the four 3D CNNs under the different size of the training dataset.

Model	The size of training set (%)	Recall/%	DSC/%	Jaccard/%	HD/%
3D UNet	100	—	—	—	—
	50	−1.55	0.05	0.07	−6.16
	25	−0.67	−0.29	−0.55	−13.96
VNet	100	—	—	—	—
	50	−1.70	−1.46	−2.72	−18.04
	25	−3.83	−6.97	−11.54	−80.93
3D Res-UNet	100	—	—	—	—
	50	0.03	−0.93	−1.72	−4.88
	25	−2.40	−2.02	−3.67	−56.41
SegResNet	100	—	—	—	—
	50	0.37	0.11	0.17	−32.34
	25	−1.18	−0.28	−0.52	−34.67

In detail, when the size of the training set was reduced to 50% of the entire training set, the segmentation quality of the 3D CNNs was comparable on the evaluation metrics. However, when the size of the training set was reduced to 25% of the entire training set, the segmentation quality of VNet showed the most notable deterioration on recall, DSC, and Jaccard by 3.83%, 6.97%, and 11.54%, while that of the other three 3D CNNs was decreased by less than 4%. Even though the recall, DSC, and Jaccard of 3D UNet, 3D Res-UNet and SegResNet did not show a great decrease, HD of them was sharply deteriorated by 13.96%, 56.41% and 34.67%, respectively, indicating that the inadequate training dataset could severely affect the CNN-based segmentation performance.

### 3.3 Segmentation performance comparison

#### 3.3.1 Segmentation quality evaluation

Table 3 shows the segmentation performance metrics of the 3D CNNs. All the 3D CNNs achieved a mean DSC higher than 0.95 over the five-fold cross-validation, and the differences in mean DSC between groups were less than 1%. Similar to DSC, the differences in other metrics between groups were less than 1%, including recall and Jaccard index. In terms of HD, VNet (7.72 ± 1.89) and 3D Res-UNet (8.56 ± 2.28) performed better than SegResNet (10.74 ± 5.41) and 3D UNet (15.96 ± 8.71). The difference between VNet and 3D Res-UNet is within 10%, while the difference between VNet and 3D UNet as well as SegResNet is beyond 30%.

**TABLE 3:** Cross validation results of different models.

Networks	SEN/%	DSC	Jaccard	HD	Time/s
3D UNet	95.72 ± 0.15	0.954 ± 0.044	0.912 ± 0.007	15.96 ± 8.71	1.13 ± 0.58
VNet	95.45 ± 0.89	0.958 ± 0.004	0.912 ± 0.007	7.72 ± 1.89	2.14 ± 1.17
3D Res-UNet	95.65 ± 0.67	0.961 ± 0.004	0.920 ± 0.009	8.56 ± 2.28	0.10 ± 0.04
SegResNet	96.52 ± 0.59	0.957 ± 0.005	0.919 ± 0.008	10.74 ± 5.41	0.28 ± 0.15

**FIGURE 7:**
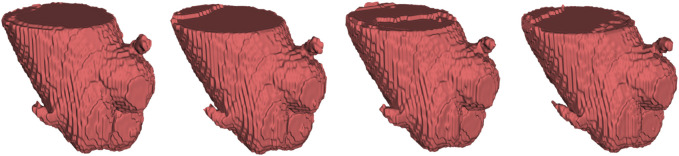
From left to right, automatic 3D reconstruction visualization results based on 3D UNet, VNet, 3D Res-UNet and SegResNet, respectively.

To provide the details of 3D deviations between CNN-based segmentation and ground truth, 3D surface reconstruction using the marching cubes algorithm based on Python platform and VTK library was conducted to obtain the 3D reconstruction models from CNN-based segmentation and ground truth. Specifically, due to the bilinear interpolation conducted to compress the data in image pre-processing, it is essential to re-sample the size of CNN-based automatic segmentation to the original image size before 3D reconstruction. After automatic 3D reconstruction, we processed the reconstruction models from CNN-based segmentation and ground truth by surface smoothing (Figure 7). To evaluate the 3D deviations between the 3D reconstruction models from CNN-based segmentation and ground truth, four metrics widely used in 3D model deviation analysis were evaluated in this study, including maximum distance, average distance, standard deviation (STD), and root mean square (RMS) value (Table 4). It was found that the segmentation results of 3D UNet have the largest maximum and averaged deviation from the ground truth compared with other evaluated 3D CNNs, which might be attributed to the lack of residual block.

**TABLE 4:** 3D deviation analysis based on automatic 3D reconstruction results.

Networks	Max distance/mm	Average distance/mm	STD/mm	RMS/mm
3D UNet	+2.6149/−2.6207	0.195	0.589	0.631
VNet	+2.5002/−2.6161	0.159	0.541	0.576
3D Res-UNet	+2.5527/−2.6121	0.152	0.526	0.557
SegResNet	+2.5616/−2.5238	0.174	0.570	0.607

**FIGURE 8:**
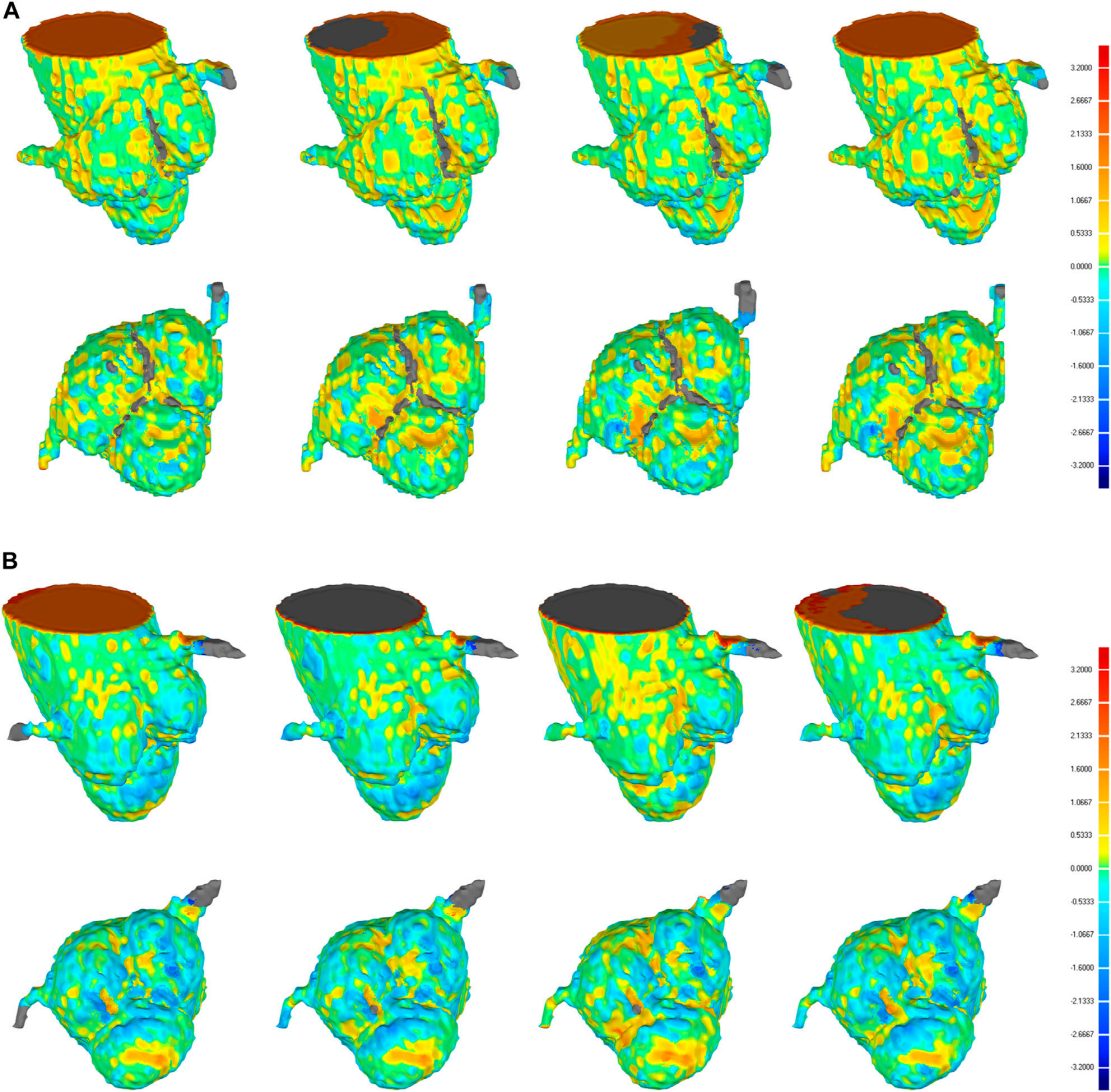
3D deviation graph of 3D automatic reconstruction models of manual segmentation and 3D automatic reconstruction models of automatic segmentation for two cases with higher DSC **(A)** and lower DSC **(B)** in the validation set. Four each case, from left to right, we show the 3D deviation map of 3D reconstruction models of manual segmentation and 3D reconstruction models of automatic segmentation based on 3D UNet, VNet, 3D Res-UNet, SegResNet, respectively. The above is a side perspective, the below is a bottom view.

**FIGURE 9:**
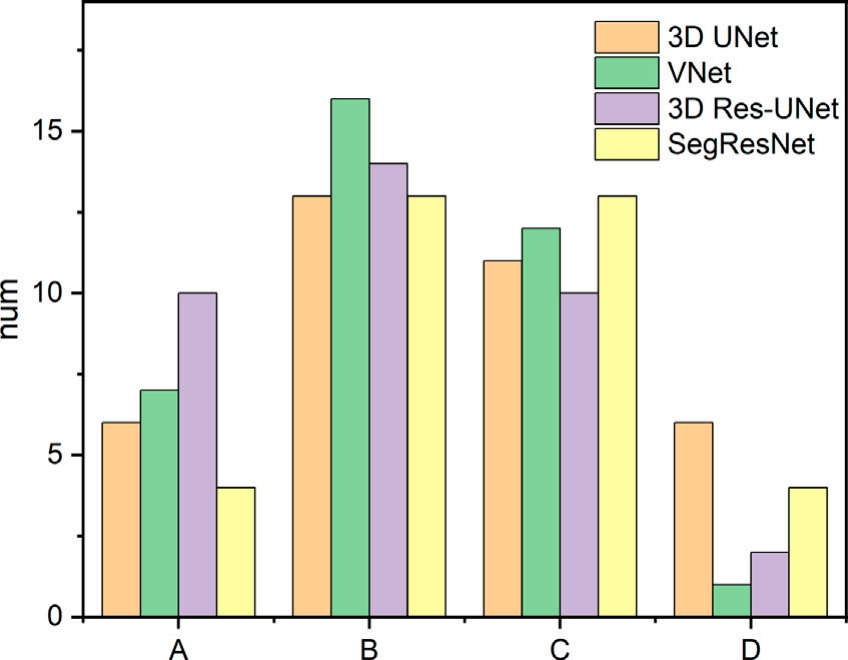
Statistics of the large deviation local position of automatic 3D reconstruction based on the four CNNs in the validation set. A, B, C, and D represent the ascending aorta, coronary arteries, aortic valve and the bottom of the aortic root, respectively.

Furthermore, the results from this study also suggested that even though the CNN-based segmentation results have the same DSC and HD, there is no necessary connection between the 3D deviation locations and classical evaluation metrics. In some cases of high DSC (>0.94) and low HD (<7), a large deviation was observed at the location of interest, including the aortic valve and the bottom of the aortic root, which is crucial for pre-operative morphological and the subsequent functional assessment of TAVR (Figure 8A). Therefore, it is incomplete and limited to evaluate the segmentation performance of 3D CNNs on the aortic root solely relying on the classical metrics. In response to this problem, a statistical analysis of the maximum deviation location was performed based on the 3D deviation cloud between the CNN-based segmentation and ground truth, including ascending aorta, coronary arteries, aortic valve, and the bottom of the aortic sinus (Figure 9). It indicated that different 3D CNNs exhibited different tendencies in the four prone deviation local positions. Despite 3D Res-UNet and VNet being more prone to conduct a large deviation at ascending aorta and coronary arteries, they performed better at the bottom of the aortic root compared with 3D UNet and SegResNet, which would be more accurate for pre-operative morphological and functional assessment of TAVR.

#### 3.3.2 Segmentation efficiency evaluation

Regarding segmentation efficiency, compared with manual segmentation, all the evaluated CNN-based average segmentation time consumed was reduced by more than 500 times from 26 min to several seconds (Table 3). 3D Res-UNet (0.1s) and SegResNet (0.28s) performed the faster segmentation time compared with that of 3D UNet (1.13s) and VNet (2.14s). Among these, the average automatic segmentation time of 3D Res-UNet was significantly shorter than that of other 3D CNNs (*p* < 0.001), which are 91.2%, 95.3%, and 64.3% faster than 3D UNet, VNet and SegResNet, respectively. Therefore, 3D Res-UNet shows greater potential for real-time pre-operative morphological assessment of TAVR among the evaluated 3D CNNs.

## 4 Discussion

In this study, the comprehensive performances of the four popular segmentation-dedicated 3D CNNs for the aortic root segmentation were quantitively evaluated. To the best of our knowledge, this is the first study focusing on the comprehensive comparison of CNN-based aortic root segmentation performance for the pre-operative assessment of TAVR. The automatic segmentation quality and efficiency results suggest that 3D Res-UNet is the most accurate and efficient 3D CNN architecture, which could provide a rapid and reliable guarantee for pre-operative morphological assessment and subsequent functional assessment of TAVR.

### 4.1 Image pre-processing

This study introduced a series of image pre-processing before training and validating 3D CNNs. Firstly, due to statistical uncertainty in all physical measurements, the inevitable noise is introduced in CT images (Diwakar and Kumar, 2018). Therefore, the median filter was conducted in the image pre-processing to improve the image quality in this study which has been demonstrated to help improve the lymph segmentation performance effectively (Zhang et al., 2021). In addition, due to the wide range of Hounsfield Unit (HU) in CT images from −1024 to 3071, it is necessary to enhance the contrast between the background and the target area by image intensity normalization, which has been proven to be beneficial to improve the segmentation performance based on CNNs (Jacobsen et al., 2019). What’s more, subject to hardware limitations, this study conducted bilinear interpolation as the image resampling method which is widely used in data compression to reduce the image resolution and further reduce the data size. Furthermore, data augmentation has become an effective solution to the limited data problem by increasing the sufficiency and diversity of the training set, especially for acquiring limited medical images (Shorten and Khoshgoftaar, 2019). Therefore, this study conducted one of the basic image augmentation methods by randomly cropping into four sub-volumes which has been proven to help reduce overfitting and the error rate of CNN models (Krizhevsky et al., 2017).

### 4.2 The selection of sub-volume size

Several studies have suggested that choosing an adequate sub-volume size in the training of 3D CNN is one of the essential factors in achieving a good segmentation result (Sabottke and Spieler, 2020). However, sub-volume size selection is a tradeoff between computation resource, time, and accuracy. In the research field of CNN-based aorta segmentation, Ravichandran et al. deployed 64 × 64 × 64 pixels sub-volume training on three NVIDIA TITAN V GPUs with 36 GB RAM (Ravichandran et al., 2019; Rukundo, 2023). Fan et al. cropped a sub-volume size of 48 × 128 × 128 pixels with a GTX 1080Ti GPU (Fan et al., 2019). To explore the suitable sub-volume size for our study, the pre-tests of the impacts of sub-volume size on 3D CNN performance were conducted. Although the pre-tests showing a larger sub-volume size would result in better segmentation quality, it also demands a faster GPU and larger RAM. This result agrees well with the previous studies (Sabottke and Spieler, 2020). Thus, subject to hardware limitation, the sub-volume size of 128 × 128 × 128 pixels was selected in this study.

### 4.3 The selection of training set size

In general, DL models represented by 3D CNNs require a large amount of training data to effectively learn the target task (Ghadimi et al., 2021). However, due to the scarcity of annotated medical images, it is hard to build a large dataset at the initial stage of developing such 3D CNNs in the field of medical image segmentation. Therefore, it is significant to investigate the CNN-based segmentation performance of the aortic root under the small sample size. Due to that the selecting of an appropriate dataset size for 3D CNNs is still a challenging open problem (Cheung et al., 2021; Nu˜nez-Garcia et al., 2019), this study conducted a sensitivity analysis to explore the impact of the training set size on the CNN-based segmentation performance of the aortic root.

The results show that the training performance and segmentation performance of VNet is most sensitive to the size of the training dataset, which might be due to its more network parameters caused by the larger convolutional kernel size (Table 1), further leading to the need for more training data to guarantee the segmentation performance. Compared with VNet, even though the recall, DSC and Jaccard of 3D UNet, 3D Res-UNet and SegResNet did not show a notable decrease with the reduction of the training dataset, HD of them was sharply deteriorated, which might indicate that less training data could severely worsen the capacity of 3D CNNs to capture complex boundary details in the aortic root segmentation. Therefore, when selecting a suitable 3D CNN under small samples, it should be considered that CNN models with higher complexity could bring the problem of reliance on large datasets, despite their excellent learning ability.

### 4.4 Segmentation performance

#### 4.4.1 Segmentation quality

##### 4.4.1.1 Classical metrics analysis

Segmentation quality is the most important aspect in evaluating CNN-based automatic segmentation. Several classical segmentation quality assessment metrics have been proposed, including recall, DSC, Jaccard index, and HD.

Among the metrics, similarity coefficients (SCs), such as DSC and Jaccard index, are the essential spatial-based metrics representing the overlap ratio between segmentation results and ground truth. As one of the classical SCs, DSC was widely used to evaluate image segmentation quality, which is traditionally considered positively correlated with segmentation quality. However, its threshold of distinguishing good and poor segmentation quality varied by several factors, including segmentation objects, CNN architectures, image quality, and dataset size. In the field of cardiovascular segmentation, a DSC of 0.7 has been well-accepted as the segmentation quality threshold (Robinson et al., 2018; Robinson et al., 2019; Hann et al., 2021). In this study, all the evaluated 3D CNNs achieved a mean DSC higher than 0.95 over the five-fold cross-validation, and the differences in mean DSC between groups were less than 1%. This result is comparable with the previous studies focused on aorta segmentation based on private CT datasets (Zou et al., 2004; Fan et al., 2019; Ravichandran et al., 2019; Macruz et al., 2022; Sieren et al., 2022), which has an average DSC of 0.917 ± 0.054 (Table 5). In addition, the Jaccard index derived from this study also agrees well with the related study (Fan et al., 2019). The comparison results demonstrated that the CNN-based automatic segmentation framework established in this study was essentially reliable.

**TABLE 5:** Comparison of the segmentation performance of this study with the previous studies.

Study	Networks	DSC	Jaccard	HD
This Study	3D Res-UNet	0.961 ± 0.004	0.920 ± 0.098	8.56 ± 2.28
Macruz et al. (2022)	3D UNet	0.920	\	\
Sieren et al. (2022)	3D UNet	0.950	\	8.00
Ravichandran et al. (2019)	3D Inception UNet	0.838	\	\
Fan et al. (2019)	Attention DRN	0.960	0.928	\

However, a single metric (DSC) cannot comprehensively reflect the segmentation quality. Therefore, HD is conducted to evaluate the segmentation quality based on the spatial distance that can quantitively indicate the largest error between the boundary of CNN-based segmentation and ground truth. HD is generally sensitive to boundary morphology, which makes it suitable for evaluating cases where the complex boundary is of interest (Taha and Hanbury, 2015). Our results showed that the average HD of 3D UNet (15.96 ± 8.71) is notably higher than that of VNet (7.72 ± 1.89), 3D Res-UNet (8.56 ± 2.28), and SegResNet (10.74 ± 5.41), which suggested that the residual block in the latter three 3D CNNs contributed to the better capture of complex boundary details in the aortic root segmentation. This result is consistent with the theoretical analysis of the function of the residual block (He et al., 2016).

##### 4.4.1.2 3D deviation analysis

Although the above classical metrics are widely used for segmentation quality assessment, they are based on the overall morphological characteristics, which cannot reflect the important geometric details for pre-operative morphological and the subsequent functional assessment of TAVR. Therefore, the 3D deviation analysis was carried out to demonstrate the detailed local errors between CNN-based segmentation and ground truth intuitively. As shown in Figure 9, all the evaluated 3D CNNs were prone to large deviations in the ascending aorta and coronary arteries due to the absence of anatomical boundaries (Figure 8B). In addition, the thin structures and ambiguous boundaries of the aortic valve are difficult to distinguish in CTA images, making it also difficult for CNNs to accurately segment (Figure 8A). Therefore, all the CNN-based segmentation are prone to large deviations in the ascending aorta, coronary arteries, and aortic valve.

Even though the deviation at the distal ends of the aortic root, such as the ascending aorta and coronary arteries, does not affect the pre-operative morphological assessment of TAVR, the segmentation quality of the aortic valve and the bottom of the aortic root is critical for successful TAVR. Therefore, compared with 3D UNet and SegResNet, 3D Res-UNet and VNet performed better at the aortic valve and the bottom of the aortic root, which would be more accurate for the pre-operative morphological and functional assessment of TAVR.

#### 4.4.2 Segmentation efficiency

In the cardiovascular field, manual segmentation is labor-intensive and time-consuming. The segmentation time varies from minutes to hours for different segmented objects, which limits the clinical applications of patient-specific analysis that rely on image segmentation, especially for TAVR which requires rapid preoperative assessment (Byrne et al., 2016).

In our study, benefitting from the improvement of the hardware platform, all 3D CNNs shorten the segmentation time by more than 500 times compared with the manual segmentation time, which agrees well with the related study in order of magnitude (Bratt et al., 2019). Among these 3D CNNs, the average segmentation time of 3D Res-UNet and SegResNet is less than 1 s. VNet uses the largest convolution kernel size which tends to be disproportionally expensive in terms of computational cost, resulting in the longest segmentation time (Szegedy et al., 2016). 3D UNet uses max pooling layers as the traditional up/down sampling function enlarging the model size and memory occupation which may lead to a longer segmentation time (Ayachi et al., 2020). In comparison, both 3D Res-UNet and SegResNet use the smaller convolutional kernel size and convolution operation instead of max pooling layers as the up/down sampling function which greatly reduces the segmentation time across orders of magnitude.

### 4.5 Limitation

Although the results of this study are promising, there are some limitations. Firstly, although this study provides a relatively comprehensive assessment of the segmentation performance of the 3D CNNs under the small dataset, it should be noted that the dataset of this study was collected from a single clinical center and the same scanner vendor, which limits the generalizability across multiple centers and scanner vendors (Campello et al., 2021). In addition, this study only used the annotation results of one annotator as a benchmark and did not consider the differences between different annotators. The next step is to compare the results of the same sample annotated by different annotators. Furthermore, FEA and CFD simulation will be conducted to evaluate the influence of CNN-based segmentation results on the mechanical and hemodynamic environment for accurate patient-specific preoperative functional assessment of TAVR.

## 5 Conclusion

The segmentation performances of four segmentation-dedicated 3D CNNs on the aortic root segmentation were evaluated in this study. Although 3D Res-UNet and VNet are evenly matched in the aspect of classical segmentation quality metrics and 3D deviation location of interest analysis, 3D Res-UNet is the most efficient CNN architecture which provides a rapid and reliable guarantee for pre-operative morphological assessment and subsequent functional assessment of TAVR.

## Data Availability

The original contributions presented in the study are included in the article/supplementary material, further inquiries can be directed to the corresponding authors.
